# Pre-treatment metastatic growth rate is associated with clinical outcome in patients with metastatic renal cell carcinoma treated with nivolumab

**DOI:** 10.1186/s12894-023-01248-z

**Published:** 2023-06-10

**Authors:** Soichi Matsumura, Taigo Kato, Yuma Kujime, Hiroaki Kitakaze, Kosuke Nakano, Sachiko Hongo, Iwao Yoshioka, Masayoshi Okumi, Norio Nonomura, Shingo Takada

**Affiliations:** 1 Department of Urology, Osaka Police Hospital, Osaka, Japan; 2 Department of Urology, Osaka University Graduate School of Medicine, 2-2 Yamadaoka, Suita, Osaka 565-0871 Japan

**Keywords:** Metastatic growth rate, Metastatic renal cell carcinoma, Nivolumab

## Abstract

**Background:**

Immune checkpoint inhibitors (ICIs) have been approved for the treatment of metastatic renal cell carcinoma (mRCC). However, the response rate is still limited, and it is urgent to pursue novel and concise markers of responses to ICIs that allow the determination of clinical benefits. Recently, it was reported that the metastatic growth rate (MGR) is an independent factor associated with clinical outcome for anticancer therapy in some types of cancer.

**Methods:**

We investigated pre-treatment MGR before starting nivolumab for mRCC patients between September 2016 to October 2019. In addition, we examined clinicopathological factors including MGR and analyzed the correlation between pre-treatment MGR and clinical efficacy of nivolumab.

**Results:**

Of all patients, the median age was 63 years (range, 42–81), and the median observation period was 13.6 months (range, 1.7–40.3). Twenty-three patients and sixteen patients were classified as the low and the high MGR group, respectively, with the cutoff value of 2.2 mm/month. Progression-free survival (PFS) and overall survival (OS) were significantly better in patients in the low MGR group (*p* = 0.005 and *p* = 0.01). Importantly, in multivariate analysis, only the high MGR was significantly associated with a decrease of PFS (Hazard ratio (HR): 2.69, *p* = 0.03) and OS (HR: 5.27, *p* = 0.02).

**Conclusions:**

Pre-treatment MGR may serve as the simple and valid indicator obtained from imaging studies, and the prominent surrogate marker associated with OS and PFS in mRCC patients treated with nivolumab.

## Background

Renal cell carcinoma (RCC) accounts for approximately 2–4% of all types of cancer worldwide [1]. The 5-year disease-specific survival rate was about 50% in 1975–1977 and improved to 75% in 2010–2015 [2]. However, almost 30% of RCC patients still present with mRCC at the initial diagnosis while another 30% develop metastasis later during the course of the disease, leading to the introduction of subsequent therapies.

Recently, the treatment of mRCC has changed dramatically with the introduction of immune checkpoint inhibitors (ICIs) [3, 4]. Among them, nivolumab, anti-programmed cell death protein 1 (PD-1) inhibitor, has been widely used for mRCC from the late 2010s, as evidenced by promising results in Checkmate 025 trial [4]. However, nivolumab has a limited response rate of 23%, and the majority of patients still not benefit from the treatment [4, 5].

To date, several types of biomarkers such as the expression of programmed death-ligand 1 (PD-L1) in cancer tissues have been proposed to have a significant impact on the clinical response to nivolumab [6]. However, these markers basically focus on the tumor state at the time of diagnosis and sometimes produce contradictory outcomes since tumors are generally subjected to drug-imposed selective pressure before the introduction of ICIs. To overcome this limitation, it is urgent to pursue novel and concise indicators of responses to ICIs that predict clinical benefits.

In the last decade, tumor growth rate (TGR) has been of increasing interest to evaluate response to anticancer therapy [7]. While Response Evaluation Criteria in Solid Tumors (RECIST) evaluation does not take into account the dynamics of the tumor prior to treatment, TGR provides a dynamic and quantitative assessment of tumor kinetics. However, it is necessary to calculate the sum of the longest diameter of several target metastases for measuring TGR, which is complicated to calculate and difficult to use in clinical practice.

In the present study, for simple and rapid method to predict clinical response to nivolumab in mRCC patients, we focused on pre-treatment metastatic growth rate (MGR), that is a simple measurement of tumor growth rate for the single largest metastasis.

## Materials and methods

### Patient

We retrospectively investigated the data of 39 mRCC patients who received nivolumab as a second-line or later-line therapy between September 2016 to October 2019 at Osaka Police Hospital and Osaka University Hospital. Patients treated with nivolumab for less than one month were excluded to accurately assess the effect of nivolumab. To measure the size of metastases, patients with metastases whose size could not be measured by imaging tests were excluded. For each patient, we collected baseline demographic and clinical data including age, gender, International Metastatic Renal Cell Carcinoma Database Consortium (IMDC) risk group, the history of prior nephrectomy, and sites of metastasis. Tumor assessments by computed tomography (CT) or magnetic resonance imaging (MRI) were performed with the use of RECIST version 1.1, at screening and every 2–3 months from the date of starting nivolumab. Patients with radiographic imaging at baseline (T(0)) prior to nivolumab administration and at pre-baseline (T(− 1)) were included in the study.

The study was approved by the Institutional Review Board of each institution (approval number 1119 in Osaka Police Hospital and 018-0003 in Osaka University Hospital) and was conducted in accordance with the Declaration of Helsinki.

### Treatment

Nivolumab was administered by intravenous infusion at a dose of 3 mg/kg or 240 mg/body every 2 weeks. PD-L1 status was not evaluated. Patients received therapy until either disease progression or unacceptable toxicity presented.

### The measurement of metastatic growth rate (MGR)

For each patient, the largest metastasis at baseline was determined using the longest diameter (D) in the axial plane. If the largest diameter was lymph node metastasis, the shortest axial diameter was used. The measurement of MGR was performed as described previously [8]. Briefly, the absolute metastatic growth in millimeters (mm) was determined as the difference between the diameter of the largest lesion at the baseline staging (D(0)) and at the pre-baseline staging (D(− 1)). This difference was divided by the number of days elapsed between pre-baseline staging and baseline staging (t), and the resulting value was multiplied by 30.4375 days to obtain the metastatic growth rate per month. The following equation summarizes this relation:

$$MGR = \frac{{D\left( 0 \right) - D\left( { - 1} \right)}}{t}\; \times \;30.4375\;({\text{mm}}/{\text{month)}}$$

**Fig. 1:**
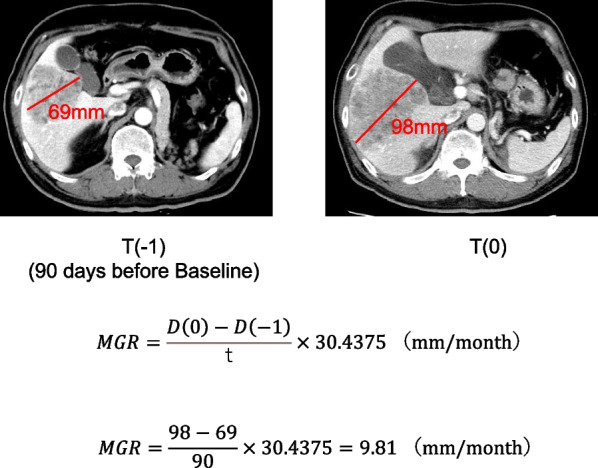
Example of MGR calculation. This patient had a liver metastasis with a maximum diameter of 98 mm prior to nivolumab administration, and a CT scan was performed 90 days earlier, which showed a diameter of 68 mm. The MGR was calculated to be 9.81 mm/month

A typical example was shown in Fig. 1. MGR was measured in the metastases with the largest diameter from imaging studies immediately before nivolumab administration.

The area under the receiver operating characteristic (ROC) curve was used to evaluate the predictive efficiency of MGR. A superior cutoff point was defined as the point on the ROC curve with a Youden index. In the present study, the cutoff value for MGR was set at 2.2 mm/month. We defined high and low MGR as patients with MGR greater than 2.2 mm/month, and less than 2.2 mm/month, respectively. As a result, high and low MGR consisted of 16 and 23 cases.

OS was defined as the time from starting nivolumab until death due to any cause or end of follow-up, whereas PFS was defined as the time from starting nivolumab until progression or death due to mRCC or end of follow-up.

### Statistical analysis

In two groups, PFS and OS were estimated using the Kaplan–Meier method and differences between two groups were assessed using the log-rank test. We performed univariate and multivariate analyses of gender, age, IMDC risk classification, baseline neutrophil to lymphocyte ratio (NLR), baseline CRP, the number of prior treatments, and MGR, and examined their associations with PFS and OS. Baseline NLR and CRP levels were categorized into two groups (above vs. below median).

In multivariate analyses for PFS and OS, the Cox proportional hazards regression model was used. Categorical variables were compared using two-sided Fisher’s exact test. For all analyses, differences were considered to be significant at *p* < 0.05. All statistical analyses were conducted in JMP-software version 14.0 (SAS Institute, Cary, NC, USA).

## Results

### Patient characteristics

The baseline characteristics of 39 patients are shown in Table 1. Patients had a median age of 63 years (42–81 years). In this study, 29 patients were men and 10 were women. IMDC risk criteria showed 9 patients (23%) with a favorable risk, 23 patients (59%) with an intermediate risk, and 7 patients (18%) with a poor risk. The median NLR and CRP level was 3.26 (0.3–14.91) and 0.81(0.04–15.51) mg/L, respectively. The most common evaluated lesion was the lung in ten cases (25.6%). The next most common lesions were lymph nodes in seven cases (17.9%) and bone in five cases (12.8%) (Table 1).

**Table 1:** Patient characteristics

		Total cohort	Low MGR (≤ 2.2 mm/month)	High MGR (> 2.2 mm/month)	*P* value
No. of patients		39	23	16	
Age(years)	Median(IQR)	63(42–81)	63(42–80)	64(46–81)	0.41
Sex	Male	29	18	11	0.71
	Female	10	5	5	
Metastatic lesion	Lung	10	3	7	
	Lymph nodes	7	7	0	
	Bone	5	3	2	
	others	17	10	7	
Previous nephrectomy	Presence	37	21	16	0.50
	Absent	2	2	0	
Pathological outcome	Clear cell renal cell carcinoma	35	21	14	
	Papillary renal cell carcinoma	3	1	2	
	Collecting duct carcinoma	1	1	0	
IMDC risk	Favorable	9	8	1	0.01
	Intermediate	23	14	9	
	Poor	7	1	6	
No. of previous systemic therapies	2nd Line	23	14	9	1
	≥ 3rd Line	16	9	7	
Prior treatment	Sunitinib	12	8	4	
	Pazopanib	7	4	3	
	Axitinib	5	3	2	
	Everolimus	5	3	2	
	Avelumab + Axitinib	3	0	3	
	others	7	5	2	
Posterior treatment	Best Supportive Care	11	4	7	
	Axitinib	9	6	3	
	Sunitinib	4	0	4	
	Everolimus	1	1	0	
	others	14	12	2	
Baseline NLR	Median(IQR)	3.26(0.30–14.9)	2.48(0.30–6.84)	4.33(1.69–14.9)	0.046
Baseline CRP (mg/L)	Median(IQR)	0.81(0.04–15.5)	0.47(0.04–7.54)	1.35(0.06–15.5)	< 0.01
Baseline MGR (mm/month)	Median(IQR)	1.99(0.11–25.0)	0.95(0.11–2.15)	4.46(2.22–25.0)	< 0.01

IMDC, International Metastatic Renal Cell Carcinoma Database Consortium; NLR, neutrophil-to-lymphocyte ratio; CRP, C-reactive protein; MGR, Metastatic growth rate

**Fig. 2:**
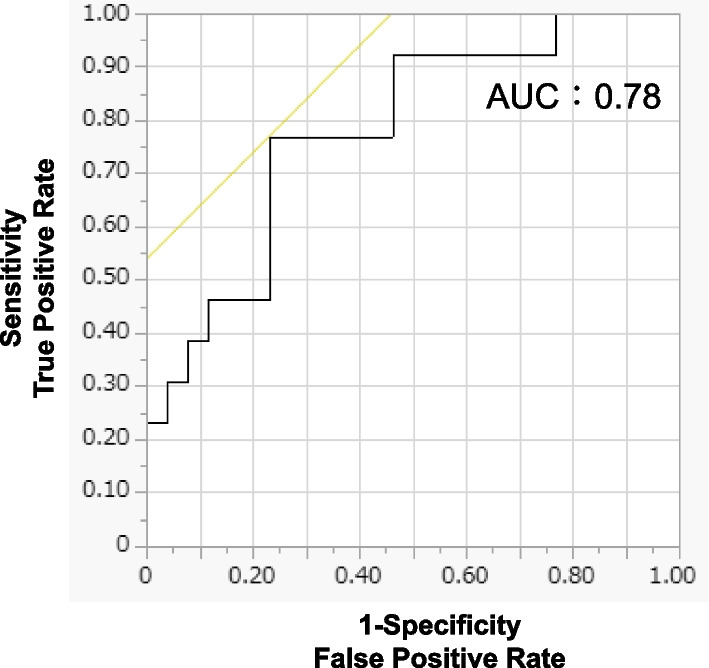
The ROC curve of pre-treatment MGR for 39 patients

Figure 2 displays the ROC curve of MGR in 39 patients for OS. As the cut-off value of MGR was set as 2.2 mm/month with the Youden index of 0.5385, 23 patients were classified as low MGR, and 16 patients were classified as high MGR. No significant difference was found among two groups for variables including age, sex, previous nephrectomy, and number of previous systemic therapies, whereas there were significant differences in IMDC risk, baseline NLR and baseline CRP between two groups (Table1).

### Pre-treatment MGR is significantly associated with better clinical outcome and predicts the response to nivolumab

**Fig. 3:**
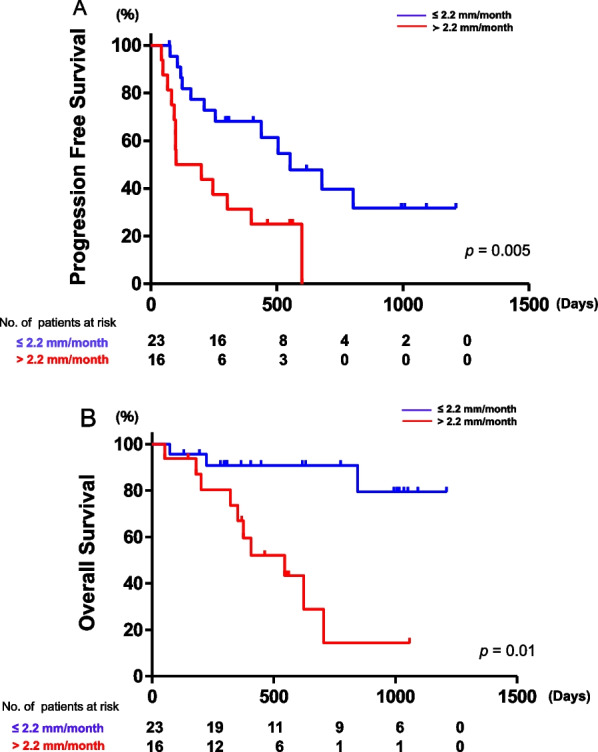
**A** Progression-free-survival curves and **B** overall survival curves based on MGR

The median PFS for the entire cohort was 9.9 months. As shown in Fig. 3A, the median PFS in the low and high MGR groups was 18.2 months and 4.9 months, respectively, which showed a significant difference between these two groups (*p* = 0.005). The median OS for the entire cohort was 13.4 months, and the median OS in the low MGR group was not reached, whereas the median OS in high MGR group was 17.9 months, leading to a significant difference between these two groups (*p* = 0.01) (Fig. 3B).

Interestingly, in univariate analysis, IMDC risk classification and pre-treatment MGR were significantly associated with an increase of OS (Table 3), whereas pre-treatment MGR was the only variable that was significantly associated with better PFS (Table 2). Multivariate analysis also demonstrated that pre-treatment MGR was the only independent prognostic factor for both OS and PFS (*p* = 0.02 and *p* = 0.03, Tables 2 and 3).

**Table 2:** Univariate and multivariate analysis for progression-free survival

PFS	Univariate analysis			Multivariate analysis		
	HR	95% CI	*p*-value	HR	95% CI	*p*-value
Age (above vs. below median)	1.14	0.52–2.51	0.74			
sex (male vs. female)	0.57	0.24–1.37	0.21			
IMDC (poor vs. fav/inter)	2.41	0.88–6.62	0.09	1.57	0.54–4.54	0.41
NLR (above vs. below median)	1.95	0.88–4.33	0.10			
CRP (above vs. below median)	1.51	0.68–3.351	0.31			
Line (3rd ≤ vs.2nd)	1.24	0.56–2.74	0.60			
MGR (2.2 ≥ vs. 2.2 <)	2.98	1.29–6.86	0.01	2.69	1.11–6.49	0.03

**Table 3:** Univariate and multivariate analysis for overall survival

OS	Univariate analysis			Multivariate analysis		
	HR	95% CI	*p*-value	HR	95% CI	*p*-value
Age (above vs. below median)	2.04	0.663–6.261	0.21			
sex (male vs. female)	0.71	0.19–2.61	0.61			
IMDC (poor vs. fav/inter)	5.37	1.63–17.8	0.006	2.53	0.72–8.94	0.15
NLR (above vs. below median)	2.82	0.86–9.23	0.09			
CRP (above vs. below median)	0.71	0.23–2.17	0.54			
Line (3rd ≤ vs.2nd)	1.09	0.35–3.33	0.89			
MGR (2.2 ≥ vs. 2.2 <)	6.99	1.84–26.6	0.004	5.27	1.25–22.4	0.02

**Fig. 4:**
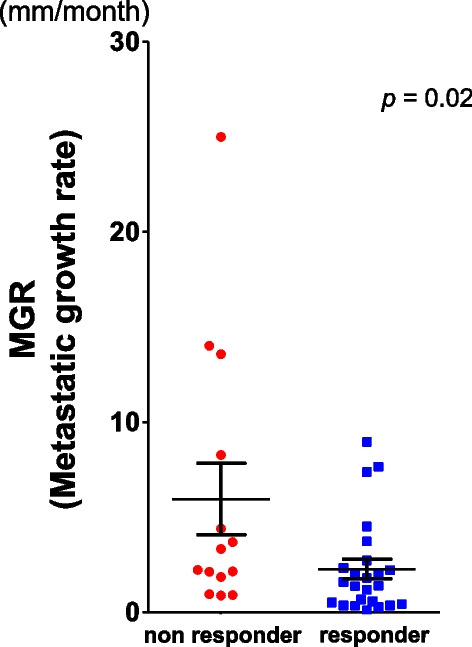
Scatter plots of responders (complete response, partial response, or stable disease (SD) for ≥ 6 months (long SD)) and non-responders (stable disease (SD) for < 6 months (short SD) or Progressive Disease). The median MGR was significantly lower in responders (*p* = 0.02)

In the present study, considering the median PFS in the clinical trial of nivolumab (Checkmate 025), we defined responders as patients with complete response, partial response, or stable disease (SD) for ≥ 6 months (long SD) [4]. As a result, responders and non-responders consisted of 25 and 14 cases, respectively. Of note, we observed that the median MGR was significantly lower in responders (*p* = 0.02, Fig. 4).

## Discussion

Despite intense progress in recent immunotherapies, the response rate of ICI monotherapy is still limited, and treatment-related adverse effects often cause discontinuation of the ICI therapy [9]. Therefore, it is urgent to identify clinical parameters to predict the response of patients to ICIs to establish the precise immunotherapeutic strategies. To date, predictive markers of response have mainly focused on PD-L1 expression of tumor tissues before the initiation of the ICI treatment [10]. However, given the intratumoral heterogeneity and discrepancy of PD-L1 expression between primary and metastatic lesions, PD-L1 expression does not reflect the entire tumor microenvironment and sometimes was not subject to reliable biomarker in mRCC [11]. Hence, in this study, we investigated tumor growth speed before starting nivolumab in patients treated with anti-PD-1 monotherapy as an alternative to immunohistochemistry, and aimed to analyze whether MGR could be concise and feasible method to predict clinical outcome of mRCC patients treated with ICIs.

In the present study, we showed that pre-treatment MGR have a significant impact on PFS and OS in mRCC patients treated with nivolumab. Our results align with the data published by Wagner et al. reported high pre-treatment MGR is an independent strong prognostic marker associated with unfavorable survival the in the context of metastatic melanoma patients. So far, several studies showed that pre-treatment TGR is an excellent calculation method in terms of determining objective response and have attracted much attention [12, 13]. For example, Grande et al. showed a significant association between TGR prior to initiation of second-line therapy and the PFS and OS including subsequent systemic therapy in mRCC patients [12]. They showed patients with a very low TGR (increase of less than 4% in the sum of the longest diameters per month) achieved long-term therapeutic effect, and have an adequate tolerability. Iacovelli et al. also showed that patients with TGR above the median value during treatment have a significantly shorter OS [14]. However, calculating TGR for multiple metastatic lesions is often time-consuming and limits sample size for analysis [15].

Importantly, according to multivariable analysis in this study, the pre-treatment MGR less than 2.2 mm/month was the only factor which was significantly associated with a better PFS as well as OS. These results clearly propose that pre-treatment MGR could be a novel and concise measure of tumor growth kinetics that independently predicts clinical outcome when compared to IMDC risk classification, baseline NLR, baseline CRP and number of prior treatments.

Some limitations should be considered when interpreting these results. First, it had a retrospective design in a small cohort with a limited follow-up duration. Further large prospective studies will be needed to confirm the credibility of pre-treatment MGR. Second, we set the MGR cutoff value at 2.2 mm/months from the obtained data. Further validation will be needed to determine if this cut-off value is appropriate for predicting the efficacy of ICIs.

## Conclusions

In conclusion, to our knowledge, we first showed that pre-treatment MGR was the simple and valid indicator obtained from imaging studies and the prominent surrogate marker associated with clinical outcome in mRCC patients treated with nivolumab.

## Data Availability

The datasets used and/or analyzed during the current study are available from the corresponding author on reasonable request.

## Abbreviations

CRP: C-reactive protein
CT: Computed tomography
HR: Hazard ratio
ICIs: Immune checkpoint inhibitors
IMDC: International metastatic renal cell carcinoma database consortium
KPS: Karnofsky performance status
MGR: Metastatic growth rate
mRCC: Metastatic renal cell carcinoma
MRI: Magnetic resonance imaging
NLR: Neutrophil to lymphocyte ratio
OS: Overall survival
PD: Progressive disease
PD-1: Programmed cell death protein 1
PD-L1: Programmed death-ligand 1
PFS: Progression free survival
RCC: Renal cell carcinoma
RECIST: Response evaluation criteria in solid tumors
ROC: Receiver operating characteristic
SD: Stable disease
TGR: Tumor growth rate
